# THE RELATIONSHIP BETWEEN INTERNET USE AND LIFE SATISFACTION IN LATER LIFE: A FOCUS ON THIRD-LEVEL DIGITAL DIVIDE

**DOI:** 10.1093/geroni/igad104.3655

**Published:** 2023-12-21

**Authors:** Ern Chern Khor, Moon Choi

**Affiliations:** KAIST, Daejeon, Republic of Korea; KAIST, Daejeon, Republic of Korea

## Abstract

The third-level digital divide highlights how social factors can shape disparities in real-life experiences derived from digital use. This study aims to examine how different types of internet use are related to the life satisfaction of older adults with varying health and wealth statuses through the lens of the third-level digital divide. The data for this study came from the 2022 Korea Digital Divide Survey. A sample of 1,165 older adults aged between 65 and 96 years old was grouped into four categories based on their health and wealth statuses, considering that these two factors tend to shape the social positions of older adults in Korean society. Life satisfaction was measured using 5 items from the 4-point Satisfaction with Life Scale (SWLS), while different types of internet use were accessed with 13 dichotomous items. The findings showed that a significant positive effect of internet use was observed only among older adults with good health status, indicating a stronger effect for those with good wealth status. Particularly, online public services (d=1.41, p< 0.001), cloud services (d=1.35, p< 0.001), and social networking services (d=1.27, p< 0.001) exhibited significant effects. No significant effect was observed for older adults with poor health status, except for online entertainment content, which was found to be significantly related to the life satisfaction of those with low wealth status (d=0.87, p< 0.01). The study findings imply diversity in terms of internet use and its association with life satisfaction, while highlighting social disparities.

